# Predicting congenital heart defects: A comparison of three data mining methods

**DOI:** 10.1371/journal.pone.0177811

**Published:** 2017-05-24

**Authors:** Yanhong Luo, Zhi Li, Husheng Guo, Hongyan Cao, Chunying Song, Xingping Guo, Yanbo Zhang

**Affiliations:** 1 Department of Health Statistics, School of Public Health, Shanxi Medical University, Taiyuan, Shanxi Province, People’s Republic of China; 2 School of Computer and Information Technology, Shanxi University, Taiyuan, Shanxi Province, People’s Republic of China; 3 Population and Family planning Commission of Shanxi province, Taiyuan, Shanxi Province, People’s Republic of China; Nanjing Normal University, CHINA

## Abstract

Congenital heart defects (CHD) is one of the most common birth defects in China. Many studies have examined risk factors for CHD, but their predictive abilities have not been evaluated. In particular, few studies have attempted to predict risks of CHD from, necessarily unbalanced, population-based cross-sectional data. Therefore, we developed and validated machine learning models for predicting, before and during pregnancy, women’s risks of bearing children with CHD. We compared the results of these models in a large-scale, comprehensive population-based retrospective cross-sectional epidemiological survey of birth defects in six counties in Shanxi Province, China, covering 2006 to 2008. This contained 78 cases of CHD among 33831 live births. We constructed nine synthetic variables to use in the models: maternal age, annual per capita income, family history, maternal history of illness, nutrition and folic acid deficiency, maternal illness in pregnancy, medication use in pregnancy, environmental risk factors in pregnancy, and unhealthy maternal lifestyle in pregnancy. The machine learning algorithms Weighted Support Vector Machine (WSVM) and Weighted Random Forest (WRF) were trained on, and a logistic regression (Logit) was fitted to, two-thirds of the data. Their predictive abilities were then tested in the remaining data. True positive rate (TPR), true negative rate (TNR), accuracy (ACC), area under the curves (AUC), G-means, and Weighted accuracy (WTacc) were used to compare the classification performance of the models. Median values, from repeating the data partitioning 1000 times, were used in all comparisons. The TPR and TNR of the three classifiers were above 0.65 and 0.93, respectively, better than any reported in the literature. TPR, wtACC, AUC and G were highest for WSVM, showing that it performed best. All three models are precise enough to identify groups at high risk of CHD. They should all be considered for future investigations of other birth defects and diseases.

## Introduction

Birth defects (BD) are a major cause of infant death. Congenital heart defects (CHD) are the most common type of birth defect in China [1]. China has reported estimated incidences of 2.396, 2.882, and 4.095 CHD per 1,000 live births in 2005, 2010, and 2011, respectively. The incidence of CHD in the perinatal period is higher than the incidence of any other BD [2]. Therefore, screening groups of women with a high- risk of a CHD birth, both before and during pregnancy, is clinically important. It is also crucial to enable early intervention and treatment of these birth defects. Prediction models, with good predictive ability for identifying women whose offspring are likely to be prone to CHD, are therefore required [3]. Models that use risk factors to predict CHD can help to identify high-risk groups in the population, allowing specific intervention strategies to be targeted at particular subgroups.

Most previous studies of CHD have focused on the distributional pattern and risk factors of CHD and assessing the associations between individual exposures and birth defects. Fewer studies have assessed the predictive ability of risk factors for CHD, or other birth defects, and predictive modeling has not been widely used to predict the risk to women of their future children suffering from CHD [4–6]. Therefore, in this study, we build predictive models that attempt to identify women likely to give birth to children with CHD.

Despite its importance, CHD affect a small proportion of births, so practical CHD classification problems are imbalanced, i.e., one of the classes makes up only a small part of the dataset. The correct classification of samples from a minority class is usually of a greater value than that for the majority class, because it is more likely to change the treatment individuals will require. Predicting the outcome of CHD is therefore of practical interest and a challenging task. Most previous studies of birth defects have used case-control data, and been based on balanced samples. So far, few studies have investigated CHD (or any other birth defects) using data from a complete population [7]. Our study is the first to predict CHD risks from all the actual live births in a general population.

For unbalanced data, interest is generally concentrated on correctly classifying the “rare” class. However, commonly used classification algorithms aim to minimize the total error rate, rather than paying particular attention to the rare class, so they do not work well for unbalanced data [8]. There are two standard methods to cope with the problem of extremely unbalanced data. One solution is grounded on a cost sensitive learning method: assigning a greater penalty to misclassifications of individuals from the rare class. The other solution is using a re-sampling technique: oversampling instances of the minority class, undersampling instances of the majority class, or both [8–10].

To avoid changing the structure of the data, we incorporated class weights into the classifiers in our study [8]. On the basis of the above considerations, we applied two popular and standard classification methods for prediction in this study: weighted support vector machine (WSVM) and weighted random forests (WRF). For comparison we also applied logistic regression (Logit) as a baseline classifier [12–13]. The objective of this study was to apply WSVM, WRF and Logit to the modelling and prediction of CHD, to test the adequacy of the predictive performance of the 3 tools, and to determine which of the 3 predictive tools best identified high risk groups of women of reproductive age.

The rest of the paper is organized as follows: Section 2 describes in detail the CHD dataset, which is used in our classification experiments, and explains the modeling and prediction methods. Prediction results from the three models, along with evaluations of their performance, are presented in Section 3. We discuss and analyze the results, outline the limitations of the work, and suggest directions for future research in Section 4. In Section 5, we summarize the research findings.

## Materials and methods

### Study design and study subjects

This study is based on data provided by the Population and Family Planning Commission (PFPC) of Shanxi Province, China. They carried out a large-scale, retrospective population-based epidemiological survey of BD in 2006–2008. The data cover six counties (Pingding, Dai, Fenyang, Huairen, Zhongyang, and Jiaokou) in the Shanxi Province of China. The six counties were selected using a stratified random cluster sampling technique. Data were collected for all live infants and their mothers. The dataset contains 33831 subjects, 78 of whom were diagnosed with CHD, so it is highly unbalanced. Ethical approval, for the both the epidemiological survey and the current study, was obtained from The Human Research Ethics Committee of Shanxi PFPC. Details of the methods for selecting the six counties, PFPC subject recruitment, and data collection have been published elsewhere [11].

Questionnaires designed by the PFPC of Shanxi Province were used to collect information about mothers' demographic characteristics, family history, maternal illness histories, premarital and pre-conception health guidance, nutritional status and dietary habits, use of folic acid supplements from 6 months before until 3 months after conception, maternal illness in pregnancy, medication use in pregnancy, exposure to hazardous substances in pregnancy, and lifestyle behaviors, as well as demographic data for their children[11]. The contents of the questionnaire have been described fully elsewhere [11].

### Predictor variables

Risk factors were assessed through in-person maternal interviews. Only variables that were significant in previous analyses or had been identified as suspected risk factors for CHD were considered in this analysis. To maximize the chance to build models with good predictive abilities, we considered a broad list of nine important indicator variables: maternal age at delivery, annual per capita income, family history, maternal history of pre-conception illness, inadequate nutrition and folic acid supplementation, maternal illness in pregnancy, medication use in pregnancy, exposure to environmental risk factors in pregnancy, and unhealthy maternal lifestyle in pregnancy. Each indicator variable, except maternal delivery age(categorical variable) and annual per capita income(ordinal categorical variable), covered multiple risk factor items and was a continuous variable. These were summed to give a “total risk factor score” and reduce the dimensionality of the data[11].

After using these data preparation strategies, the final dataset, consisted of 10 variables (9 predictor variables and 1 dependent variable) and 33831 records. The risk factors contributing to each indicator are shown in Table 1. The dependent variable is a binary categorical variable with two categories: 0 and 1, with 0 denoting non-CHD and 1 denoting CHD.

**Table 1:** Description of nine indicator variables.

Indicator variable	Risk factors	Min	Max
Maternal delivery age Annual per capita income	Maternal delivery age > 30 Annual per capita income*	0 1	1 5
Family history	Parental consanguinity^#^	0	2
	Birth defects in immediate family members^#^		
	Birth defects in previous infants^#^		
Maternal previous illness history	Hepatitis^#^	0	6
	Epilepsy^#^		
	Anemia^#^		
	Diabetes^#^		
	Heart disease^#^ Spontaneous abortion^#^		
	Thyroid disease^#^		
	Other^#^		
Nutrition and folic acid supplementation	Vegetable deficiency^△^	0	5
	Meat deficiency^△^		
	Folic acid deficiency^#^		
Maternal illness	Cold^#^	0	6
	Fever^#^		
	Threatened abortion^#^		
	Reproductive tract infections^#^		
	Hyperemesis gravidarum^#^		
	Rash and fever^#^		
	Other^#^		
Medication use	Cold medicines^#^	0	7
	Antiemetic^#^		
	Antibiotic^#^		
	Antiepileptic^#^		
	Sedative^#^		
	Contraceptive^#^		
	Abortion prevention agent^#^		
	Other^#^		
Environmental exposures of risk factors	Pesticides^△^	0	6
	Chemical fertilizers^△^		
	X-rays^△^		
	Computer use^◇^		
	Pets^△^		
	Pollution source in area of residence^△^		
Unhealthy lifestyle	Periconceptional smoking^△^	0	8
	Family member smoking^△^		
	Periconceptional drinking^△^		
	Family member drinking^△^		

* 1:less than 1000 Chinese Yuan (¥); 2:1000–2000¥; 3:2000–4000¥; 4:4000–8000¥; 5: more than 8000¥

# 0:none; 1:yes

△0:none; 1:occasionally; 2: often

◇0:none; 1:<20 hour per week; 2:≥20 hour per week and <40 hour per week; 3: ≥40 hour per week

### Data partition

For extremely imbalanced data, bootstrap resamples can include few or even none of the minority class. This will result in any classifier having poor performance at predicting the minority class. One common way of solving this problem is to use a stratified bootstrap; i.e., to sample from each class separately [8].

In our study, we split the data into non-overlapping training and test sets. We randomly selected two-thirds of the data from each class to make up the training set for building the classification models, while the remaining data were used as the test set. The training samples were used to guide model development, and the test samples were used to evaluate the predictive ability of the models. Tuning parameters were identified for each method by a grid search, for each parameter separately, using a 3-fold cross validation procedure.

As a result, our data was divided into a training set of 22554 cases, and a test set of 11277 cases. The dataset contained 9 input variables. To account for model variability, and make the results more stable, the whole procedure was repeated 1000 times and comparisons of the accuracy of three classification models used the medians of the resulting distributions [12].

### Prediction models

We used three different types of classification models: support vector machines (SVM), random forest (RF) and logistic regression (Logit). Two of these methods, RF and SVM, have been widely reported and demonstrated as successful methods for classification, but to our knowledge, they are not widely used for CHD classification applications of unbalanced data. For comparison, we also applied Logit as a baseline classifier. We describe each of these classification model types below.

#### SVM

SVM attempt to find a decision surface that perfectly separates the data points into two classes. They are based on the structural risk minimization principle. SVM employ the inner product, known as the kernel function, to map the training data into higher-dimensional feature space for nonlinear classification. SVM can find a separating hyperplane that maximizes the distance from the nearest subjects and achieves maximum separation in this higher-dimensional space. The hyperplane splits the feature space into two parts, and subjects are then classified based on which side of the hyperplane they lie on.

SVM are frequently used for classifying unbalanced data by incorporating a weighting parameter to provide extra emphasis on the rare class [10]. The kernel function can have different forms, such as the linear kernel, the polynomial kernel and the radial basis function kernel. The choice of kernel can have a large effect on model outputs. In this study, we explored several kernels and obtained optimum predictive performance for the SVM with the linear kernel, using the e1071 package in the R statistical environment. Owing to the unbalanced nature of the dataset, we used a weighted SVM (WSVM) with weights equal to the reciprocal of the class proportions [12–14].

#### Random forest

RF is based upon an ensemble of unpruned decision trees, and combines their results. Each tree is grown over a bootstrap resample with replacement. Each node of each decision tree is split using a random selection of the variables. Prediction is made by aggregating the predictions of all trees by “majority vote”. There are two important parameters in RF, the number of predictive variables to randomly choose at each node for splitting (mtry) and the number of trees to grow in the forest (ntree) [14–16]. The strategies for handling unbalanced data sets in RF, parallel those for SVM. One approach is based on cost sensitive learning, and the other is based on a sampling technique. To preserve the structure of the data, we chose to use the weighted random forest (WRF) [15] and assigned a weight to each class, with the minority class given larger weight (i.e., higher misclassification cost).

We used the R package randomForest, with 500 trees (the default value) and mtry = 3, which is square root of the total number of predictor variables (again the default value). The weighting was set at one to two, as proposed by Jiangeng and Zhikun, and the node size equaled 65. All these were tuned using a grid search with 3-fold cross validation [14–16].

#### Logistic regression

The Logit models reflect the relationship between a binary or multi-class dependent variable and a series of independent variables which may be categorical, continuous or dichotomous. Logit models predict class probabilities from a linear model to by using a logit transformation [17–19]. Logit models can only result in a predicted probability of the occurrence of a specific outcome, or of being in a particular state, and not a binary classification [18]. In this study, we used the glm function available in R. In a two-class problem, probability greater than 50% would mean that the case is assigned to the class designated as ‘‘1”, and ‘‘0” otherwise. For unbalanced data, 50 percent is not an appropriate cutoff. We used the point on the ROC (Receiver Operating Characteristic) that maximized the value of the Youden index [19–20].

### Performance evaluation for the classification methods

We require a classifier that gives high prediction accuracy over the minority class, and also has reasonable accuracy for the majority class. We used several standard performance metrics, namely: true positive rate (TPR), true negative rate (TNR), accuracy (ACC), Weighted ACC (wtACC), G-mean, and the area under the curves (AUC) to assess the performance of the three different classifiers. We will define each of the performance measures in turn, after first defining true positives (TP), true negatives (TN), false positives (FP) and false negatives (FN), respectively. TP and TN are correctly classified CHD and non-CHD, respectively; FP denotes non-CHD that are misclassified as CHD; CHD incorrectly classified as controls are FN [21]. Then:

$$\begin{array}{l} \text{TPR}=\text{TP}/(\text{TP}+\text{FN})\\ \text{TNR}=\text{TN}/(\text{TN}+\text{FP})\\ \text{ACC}=(\text{TP}+\text{TN})/(\text{TP}+\text{TN}+\text{FP}+\text{FN})\\ \text{wtACC}=\text{w}\times \text{TPR}+(1\text{-w})\times \text{TNR}\\ \text{G mean}={\left(\text{TPR}\times \text{TNR}\right)}^{1/2} \end{array}$$

we used w = 0.7 to give higher weights for accuracy on the CHD prediction [22–23].

For extremely unbalanced data, where the rare class is of great interest, TPR, G-mean, AUC and wtACC are commonly considered most important.

## Results

### Maternal demographic characteristics

The average maternal age at delivery was 25 years. The percent of maternal delivery ages below 30 was 75.9%, and above or equal to 30 was 24.1%. Around 11.1% of mothers had an annual net income per capita of less than 1000 Chinese Yuan (¥); 27.2% had an annual income of 1000–2000¥; 36.3% of 2000–4000¥; 19.3% of 4000–8000¥; and 6.2% had an annual income of more than 8000¥.

### Performance comparison of the methods

We compared the classification accuracy of the three classification models namely, WSVM, Logit, and WRF. Table 2 shows the predictive performances of the classifiers for the test data set over the 1000 data partitionings.

**Table 2:** Performance comparison of the three classifiers on the CHD data.

Indicator	Classifier	Percentiles														
		minimum	0.5	2.5	5.0	10	20	25	50	75	80	90	95	97.5	99.5	maximum
TPR	WSVM	0.3800	0.4231	0.5000	0.5385	0.5769	0.6154	0.6154	0.6923	0.7308	0.7308	0.7692	0.8077	0.8077	0.8462	0.8800
	Logit	0.3800	0.4231	0.5000	0.5000	0.5385	0.5769	0.6154	0.6538	0.6923	0.7308	0.7692	0.7692	0.8077	0.8462	0.9200
	WRF	0.3800	0.4615	0.5000	0.5385	0.5385	0.5769	0.6154	0.6538	0.7308	0.7308	0.7692	0.8077	0.8462	0.8462	0.8800
TNR	WSVM	0.9200	0.9421	0.9438	0.9445	0.9453	0.9460	0.9463	0.9476	0.9491	0.9499	0.9689	0.9924	0.9933	0.9946	1.0000
	Logit	0.8000	0.8482	0.9073	0.9332	0.9564	0.9674	0.9702	0.9813	0.9868	0.9877	0.9892	0.9904	0.9913	0.9939	0.9900
	WRF	0.8800	0.8856	0.8998	0.9053	0.9116	0.9184	0.9205	0.9304	0.9396	0.9414	0.9460	0.9517	0.9546	0.9618	0.9600
ACC	WSVM	0.9200	0.9415	0.9432	0.9440	0.9447	0.9455	0.9457	0.9470	0.9485	0.9492	0.9681	0.9914	0.9924	0.9936	0.9900
	Logit	0.8000	0.8483	0.9069	0.9328	0.9558	0.9667	0.9695	0.9806	0.9860	0.9870	0.9885	0.9894	0.9903	0.9931	0.9900
	WRF	0.8800	0.8853	0.8995	0.9049	0.9113	0.9179	0.9200	0.9298	0.9389	0.9407	0.9455	0.9510	0.9537	0.9610	0.9600
WTacc	WSVM	0.5700	0.5813	0.6347	0.6611	0.6873	0.7145	0.7151	0.7681	0.7955	0.7961	0.8229	0.8493	0.8503	0.8776	0.9000
	Logit	0.5600	0.5928	0.6429	0.6471	0.6721	0.6998	0.7097	0.7512	0.7808	0.7988	0.8175	0.8343	0.8565	0.8828	0.9200
	WRF	0.5500	0.6047	0.6314	0.6518	0.6650	0.6910	0.7092	0.7413	0.7893	0.7921	0.8166	0.8410	0.8614	0.8739	0.8900
AUC	WSVM	0.6800	0.6895	0.7276	0.7435	0.7620	0.7811	0.7821	0.8187	0.8388	0.8398	0.8587	0.8770	0.8788	0.8985	0.9200
	Logit	0.6800	0.7059	0.7364	0.7449	0.7610	0.7808	0.7836	0.8149	0.8387	0.8418	0.8600	0.8758	0.8822	0.9072	0.9300
	WRF	0.6700	0.6985	0.7258	0.7375	0.7547	0.7776	0.7852	0.8170	0.8474	0.8548	0.8711	0.8916	0.9028	0.9260	0.9300
G	WSVM	0.6200	0.6341	0.6888	0.7142	0.7384	0.7628	0.7638	0.8088	0.8317	0.8326	0.8540	0.8743	0.8759	0.8970	0.9200
	Logit	0.6100	0.6468	0.6987	0.7037	0.7278	0.7542	0.7574	0.7992	0.8262	0.8292	0.8506	0.8695	0.8791	0.9052	0.9300
	WRF	0.6000	0.6583	0.6849	0.7024	0.7186	0.7431	0.7558	0.7860	0.8226	0.8266	0.8445	0.8614	0.8713	0.8912	0.9000

From Table 2, we can see that TPR ranged from a low of 0.3800 to a high of 0.8800 for WSVM and WRF, and a low of 0.3800 to a high of 0.9200 for Logit. A previous study used a 10-point cardiovascular profile score and obtained low sensitivity (0.2500 and 0.2700, respectively) for low Apgar scores and mortality when predicting the outcome of fetal CHD [24]. Using the extended basic foetal heart examination, a sensitivity of 0.4280 in the prenatal diagnosis of CHD overall has been achieved [25]. TNR ranged from a low of just above 0.8000 to a high of above 0.96 for the three models, suggesting a satisfactory result for the extremely unbalanced CHD data in our study. In a previous study, screening tests have displayed 0.7890 specificity when looking for congenital cardiac defects (CCD) at 11–14 weeks of gestation [26]. ACC ranged from a low of above 0.8000 to a high of above 0.9900 for the three classifiers in our study. The classification of neural tube defect was predicted using SVM by Wang *et al*., and the accuracy of their prediction was 0.6900 for the test dataset [27]. AUC ranged from a low of about 0.6700 to a high of about 0.9200 for the three classifiers in our study. To assess the predictive ability of established risk factors for neural tube defects, the multivariable model was used by Agopian *et al*. They obtained AUC scores for composite NTDs, spina bifida, and anencephaly of 0.5600, 0.5500, and 0.5900, respectively [7]. A previous study applied a previously developed statistical method to investigate risk prediction on sub-phenotypes of oral clefts; their results suggested subtypes of cleft lip (CL) and palate have similar genetic etiologies (AUC = 0.5720) with subtypes of CL only (AUC = 0.5890) [28]. The value of WTacc (about 0.5500–0.8900) and G (about 0.6000–0.9000) in our study were also satisfactory for this extremely unbalanced CHD data.

The prediction results of three classifiers were summarized as the median values obtained from repeating the data partitioning 1000 times for each model. The median, over the 1000 runs, values of TPR, TNR, ACC, wtACC, AUC and G for each of the three classifiers are listed in Table 3, and their interquartile range are also shown. The TPR of the different models ranged from a low of 0.6538 for the Logit and WRF to a high of 0.6923 for WSVM. TNR ranged from a low of 0.9304 for WRF to a high of 0.9813 for the Logit. The median ACC for each of the three classification models were high (0.9298–0.9806). Over 65%, 93%, and 92% of TPR, TNR and ACC, respectively, were achieved by all the models. All three methods improved the prediction accuracy of the minority class, while maintaining high specificity.

**Table 3:** Summary of model performance (median (Q1-Q3))

Model	TPR	TNR	ACC	wtACC	AUC	G
WSVM	.6923(.6154-.7308)	.9476(.9463-.9491)	.9470(.9457-.9485)	.7681(.7151-.7955)	.8187(.7821-.8388)	.8088(.7638-.8317)
Logit	.6538(.6154-.6923)	.9813(.9702-.9868)	.9806(.9695-.9860)	.7512(.7097-.7808)	.8149(.7836-.8387)	.7992(.7574-.8262)
WRF	.6538(.6154-.7308)	.9304(.9205-.9396)	.9298(.9200-.9389)	.7413(.7092-.7893)	.7986(.7714-.8284)	.7860(.7558-.8226)

From Table 3, we can see that the WSVM model achieved a classification accuracy of 0.9470 with a TPR of 0.6923 and a TNR of 0.9476. The Logit achieved a classification accuracy of 9806 with a TPR of 0.6538 and a TNR of 0.9813. WRF achieved a classification accuracy of 0.9298 with a TPR of 0.6538 and a TNR of 0.9304. Both WSVM and Logit performed better than WRF on all metrics. This shows that, while RF is a fine classification model and shows excellent performance in many applications, its performance degrades in the presence of unbalanced data.

However, WSVM and Logit are very similar in TPR, TNR and ACC. Note that WSVM tend to focus more, than the other methods, on the accuracy of the minority class, trading off accuracy in the majority class. WSVM shows higher TPR than Logit (0.6923 *vs*. 0.6538), but is worse in TNR (0.9476 *vs*. 0.9813) and ACC (0.9470 *vs*. 0.9806).

It is clinically very important to have a high TPR so that patients with a particular potentially fatal condition are properly identified. Comparing the three models shows that the SVM model has a higher sensitivity, thus correctly classifying more of the women whose offspring were prone to CHD, while also maintaining good levels of specificity. The optimized logistic regression performed well for TNR but this comes at the cost of a lower TPR [23].

For unbalanced data, we used wtACC, AUC, G-mean to further evaluate the performance of these two methods. The three metrics (Table 3) show that the model based on the WSVM outperforms the other two models (0.7681 wtACC, 0.8187 AUC, and 0.8088 G-mean). Clearly in terms of median wtACC, AUC, and G-mean, WSVM is the winner, followed by Logit, and WRF performed worst on average.

## Discussion

In this paper, we report on our research project where we developed models to predict CHD. One important feature of this research effort is the quality and large volume of the data processed in developing these class prediction models. We used all the live births of six counties in Shanxi Province, China, which is the most comprehensive source of information on CHD in ShanXi China. Although the data were extremely unbalanced, our models performed satisfactorily.

The predictive models of our study can discriminate between high and low risk. As long as the prediction value of individual was 1, individual was judged to be at high risk for CHD, and would benefit from early addition of screening and diagnosis. The previous outcome of Logit and WRF showed family history, maternal previous illness history, maternal illness, insufficiency of nutrition, and folic acid supplementation were important risk factors for CHD. The higher-risk individuals during pregnancy need to avoid the controllable risk factors. For woman preparing for pregnancy, if the prediction value was 1, she should timely to avoid the risk factors above and other risk factors, to prevent the occurrence of birth defects.

Six counties (Pingding, Dai, Fenyang, Huairen, Zhongyang, and Jiaokou) in the Shanxi Province in our study were selected based on economic levels and geographic position, and the six counties can represent other counties in the Shanxi Province. The models in our study can been used to predict CHD in other counties in the Shanxi Province, and can be used for refer for counties of other provinces.

This article compares three models, WSVM, Logit, and WRF. We evaluated their predicted performance using six metrics, and compared our results to those obtained in other studies. The results shown above demonstrate that advanced data mining methods can be used to develop models that possess a high degree of predictive accuracy. From the results on our data set, we can conclude that both WSVM and WRF perform satisfactorily. However, WSVM did better than WRF.

We are not aware of any other study that provides class prediction based on a large-scale, population based retrospective epidemiological survey for all live births, like that of these six counties of ShanXi province in China. The data was extremely unbalanced. We compared the prediction performance of WSVM, Logit and WRF to classify women, before and during pregnancy, into one of two mutually exclusive categories (CHD *vs*. non-CHD), and found that WSVM outperformed other two classifiers. We found that modern classification methods offered improved performance for classifying women before and during pregnancy. Our conclusions are strongly supported by our analysis of class accuracies for rare classes. Our results showed that all three predictive models for CHD in our study have good predictive ability.

This work differs from previous studies in four ways, giving this study four advantages:

First, while many predictive models have been developed and used for a great variety of other diseases, predictive models based on risk factors have not been widely used to predict risk for CHD or other BD [29–30]. Comprehensive studies of CHD have focused on exploring risk factors for CHD, or investigating the prevalence at live births with CHD [5,31–33]. The CHD prediction model in our study discriminates between CHD and non-CHD individuals on the basis of unbalanced data on birth defects. Our findings indicate that research focusing on developing predictive models for CHD is needed. In the present analysis, our prediction models appeared suitable for population based screening to identify women at high-risk for CHD in their offspring. Predictive models with good predictive ability can also be helpful for individual risk counseling. The availability of prediction models with good predictive ability could help with preventing future birth defects, by providing screening tools for individuals at high-risk for CHD in offspring, as well as by guiding development of intervention strategies specific to high-risk subgroups of women with single or multiple risk factors, so as to substantially decrease the risk of CHD in future pregnancies [23–24, 26–28].

Second, in comparison with the few previous CHD prediction studies, an obvious strength of this study is our method worked on unbalanced data from a large-scale, retrospective population-based cross-sectional survey [34]. CHD occurrence is a low probability event, often affected by many environmental and social factors. However CHD are a leading cause of infant deaths in developing countries, and the incidence CHD for live births was larger than that of any other birth defect in our study, a result that is in agreement with the findings of a previous study [2]. Delayed diagnosis of CHD is associated with worse preoperative condition. Screening infants with non-invasive measurement has been proposed as an aid for early detection of CHD. It is appropriate to use a risk prediction model such as ours to identify a high-risk group of CHD for further screening.

Third, the TPR and TNR of the CHD prediction model in our study compare favorably with those from previous birth defects prediction models. We conducted rigorous comparisons of the three classification methods. All three methods were shown to improve the prediction accuracy over the minority class, while maintaining high specificity. We can conclude that, for the CHD data, Logit, WSVM and WRF with appropriate parameter values outperform previously published methods [24,26–27]. One simple conclusion from our results is that WSVM produced the highest average scores on four performance metrics (TPR, WTacc, AUC, G) over our testing data sets. We believe that our CHD prediction model makes is directly applicable for use in the primary care setting.

Fourth, to avoid changing the structure of the data, we incorporated class weights into the classifiers in our study, making it cost sensitive [8]. All three methods improve the prediction accuracy of the minority class, while maintaining high specificity, so we can conclude that for the CHD data Logit, WSVM and WRF with proper parameters outperform previously published results [24,26,28].

Fifth, logistic regression only gives prediction probability, with a dichotomous variable whose values are derived from the estimated logistic probabilities. To obtain the derived dichotomous variable, a cutpoint, *c*, has to be defined [35]. The most commonly used value for *c* is 0.5. Probability greater than 0.5 would mean that the case is assigned to the class designated as ‘‘1” and ‘‘0” otherwise. The cutoff greatly influences TPR and TNR.

In a two-class problem, it is not appropriate to use 0.5 as the cutoff for unbalanced data. We selected the point on the ROC giving the maximum value for the Youden index [19] as our cutpoint. The logistic model of our study performs well, and the alternative cutoff values improved prediction performance. When focusing on predicting the class of the presence of CHD, conventional logistic regression with cutoff of 0.5 had lower predictive accuracy compared with all the other methods that we examined. Logistic regression in our study had the best predictive accuracy for predicting the presence of CHD.

There are several limitations to this study. However, these limitations should not seriously affect the predictions.

First, this study concentrated on livebirths, and did not consider terminations of pregnancies following the prenatal diagnosis of a fetal anomaly, and late miscarriages and stillbirths affected by CHD. Our data only included live births occurring in 2006–2008, and excluded stillbirths before 28 weeks, which may have accounted for a significant proportion of birth defects. We also did not differentiate between preterm birth and full-term birth in our study, a distinction that is relevant to the detection of maternal exposure to risk factors.

Second, owing to the cross-sectional and retrospective design of our study, conclusions cannot be made about cause and effect, and the results should therefore be interpreted with caution.

Third, recall bias commonly occurs in retrospective studies. Recall bias existed in this study.

Fourth, despite the large overall sample size in this study, the sample size for cases with CHD was extremely small, and this may have limited our ability to develop models within the subgroup of CHD.

## Conclusion

This work is the first study of the prediction of CHD classification based on imbalanced data. In this study, we build predictive models to discriminate women whose offspring can be expected to have CHD from those where this is less likely. In this paper, we compare the prediction performance of three classifiers when the data is unbalanced. Three methods are shown to improve the prediction accuracy of the minority class, while maintaining high specificity. We can conclude that for the CHD data Logit, WSVM and WRF with appropriate parameter values outperform the published results. We further show that WSVM is substantially better than the other two methods, and the classification performance of Logit is better than WRF. Our result has implications in assisting clinical decision making towards accurate medical prognosis. Screening CHD high-risk groups of women before and during pregnancy is highly desirable in clinical applications, and is crucial for early specific interventions for birth defects. This study suggests that the three classifiers, which are noninvasive, can be used as a screening tool for detecting CHD high-risk groups of women before and during pregnancy. In future work, it would be interesting to explore more classifiers. In recent years, most classifiers have effective variants. For example, there are certain other advanced variants of SVM, such as twin support vector machine (TSVM), fuzzy support vector machine (FSVM), generalized eigenvalue proximal support vector machine (GEPSVM) et al.[36–40]. In our future research we intend to focus on analyzing and modeling CHD classification using other advanced machine learning methods, such as advanced variants of SVM, deep learning, feedforward neural network and ensembling[22,41].

However, our data came from a survey, rather than diagnostic data, so its prediction performance may be inferior to that of diagnostic data. We note that the classifier is not expected to replace extensive CHD diagnosis. Rather, it is intended as an initial screening method that will hopefully detect high- risk groups of women in the population before and during pregnancy. Those identified by the prediction results need to be referred for further cardiovascular tests and examined by expert cardiologists [20].

## Supporting information

S1 Dataset All variables dataset. (XLS)

## Abbreviations

CHD: Congenital heart defects
SVM: Support vector machines
WSVM: Weighted support vector machine
RF: Random forest
WRF: Weighted random forest
Logit: Logistic regression
BD: Birth defects
ROC: Receiver operating characteristic
TPR: True positive rate
TNR: True negative rate
ACC: Accuracy
WtACC: Weighted accuracy
TP: True positives
TN: True negatives
FP: False positives
FN: False negatives
AUC: The area under the curves
